# Challenges and practices in promoting (ageing) employees working career in the health care sector – case studies from Germany, Finland and the UK

**DOI:** 10.1186/s12913-019-4655-3

**Published:** 2019-11-29

**Authors:** Sebastian Merkel, Mervi Ruokolainen, Daniel Holman

**Affiliations:** 1 Institute for Work and Technology, Westfälische Hochschule, Munscheidstr. 14, 45886 Gelsenkirchen, Germany; 2 Finnish Institute of Occupational Health, FI-00032 Työterveyslaitos, Finnland; 3 Department of Sociological Studies, The University of Sheffield, Western Bank, Sheffield, S10 2TN UK

**Keywords:** Ageing employees, Age management, Work ability, Working career, Health, Social care

## Abstract

**Background:**

The health and social care sector (HCS) is currently facing multiple challenges across Europe: against the background of ageing societies, more people are in need of care. Simultaneously, several countries report a lack of skilled personnel. Due to its structural characteristics, including a high share of part-time workers, an ageing workforce, and challenging working conditions, the HCS requires measures and strategies to deal with these challenges.

**Methods:**

This qualitative study analyses if and how organisations in three countries (Germany, Finland, and the UK) report similar challenges and how they support longer working careers in the HCS. Therefore, we conducted multiple case studies in care organisations. Altogether 54 semi-structured interviews with employees and representatives of management were carried out and analysed thematically.

**Results:**

Analysis of the interviews revealed that there are similar challenges reported across the countries. Multiple organisational measures and strategies to improve the work ability and working life participation of (ageing) workers were identified. We identified similar challenges across our cases but different strategies in responding to them. With respect to the organisational measures, our results showed that the studied organisations did not implement any age-specific management strategies but realised different reactive and proactive human relation measures aiming at maintaining and improving employees’ work ability (i.e., health, competence and motivation) and longer working careers.

**Conclusions:**

Organisations within the HCS tend to focus on the recruitment of younger workers and/or migrant workers to address the current lack of skilled personnel. The idea of explicitly focusing on ageing workers and the concept of age management as a possible solution seems to lack awareness and/or popularity among organisations in the sector. The concept of age management offers a broad range of measures, which could be beneficial for both, employees and employers/organisations. Employees could benefit from a better occupational well-being and more meaningful careers, while employers could benefit from more committed employees with enhanced productivity, work ability and possibly a longer career.

## Background

Based on current debates about early retirement in combination with population ageing, policy makers have shifted to a policy of late retirement [7, 18]. Recent concerns are that pension and labour market reforms as well as human resource measures aimed at delaying retirement and extending working lives are accompanied by a (re) emergence of social inequalities in late career employment and retirement transitions. In particular, high-skilled employees do not only expect to but also desire to work longer whereas others want to retire, are pushed out of the labour market or expect to be forced to work longer in order to ensure a reasonable pension [20, 21]. Preferences for early retirement have been observed to be higher among employees with demanding working conditions and lower work ability (e.g., [3]; [6] Carr et al., 2016; von Bonsdorff, Huuhtanen, Tuomi, & Seitsamo, 2009).

One can assume that the health and social care sector (henceforth HCS) will be particularly affected by policy reforms aiming at increasing retirement ages for several reasons: Firstly, the sector is characterised by a rising demand for workers. In 2010, the number of employees in this sector stood at 21.9 million in EU countries (10.3% of all sectors); in 2018, it had increased to 24.6 million (11% of all sectors) [12]. This trend holds true especially in Western European countries. For example, Germany and the UK have, besides Hungary, the highest values of vacancy rates in 2018/19 in this sector [13]. The demand for workers is exacerbated by the fact that the sector shows a high rate of part-time employment: approximately 32% of the persons in this sector work with part-time contracts [11]. Secondly, the workforce is predominantly female: between 2008 and 2013, women filled almost 80% of the new jobs in this sector [11]. Moreover, the HCS is characterised by a gender pay gap [14]. Although the pay gap has decreased in recent years, the countries in this study (Germany, Finland, and the UK) show high values compared to other European countries [14]. Thus, women in the HCS do not only have fragmented working careers [17] but also lower salaries leading to a reduction of pension income. Thirdly, the overall workforce in the HCS is older than in other sectors. The number of persons aged 50 to 74 years working in the sector has continuously increased (see Table 1). Over one third of employees in the HCS are at least 50 years old and will leave the labour market within the next 15 years [32]. However, a recent study shows that professional care workers would like to retire earlier compared to other sectors, but do not think that they will be able to do so without avoiding pension cuts ([29] Mäcken et al., 2018).

**Table 1:** Employment (total number and share) of persons aged 50 to 74 years within the HCS (thousands)

	2010	2011	2012	2013	2014	2015	2016	2017	2018
EU	6717.1 (30.3%)	7052.2 (31.2%)	7341.4 (32.1%)	7622.6 (33.1%)	8021.1 (34.0%)	8336.8 (34.8%)	8588.9 (35.4%)	8923.5 (36.0%)	9105.4 (36.2%)
GER	1337.8 (29.0%)	1425.8 (30.9%)	1505.0 (30.9%)	1575.9 (32.5%)	1680.4 (33.9%)	1789.9 (35.1%)	1899.8 (36.0%)	1993.9 (37.2%)	2072.3 (38.1%)
FIN	141.0 (37.2%)	146.1 (37.0%)	151.9 (37.2%)	150.6 (37.7%)	153.3 (38.1%)	157.8 (39.0%)	159.3 (39.0%)	156.5 (38.7%)	157.2 (37.7%)
UK	1207.4 (31.6%)	1257.4 (32.0%)	1251.4 (32.1%)	1334.1 (33.2%)	1365.3 (33.5%)	1410.1 (34.1%)	1432.8 (34. 7%)	1496.9 (35.7%)	1529.9 (36.0%)

Fourthly, the workers in the HCS are often faced with (physically and mentally) challenging working conditions such as high work load, time pressure, emotional labour, and poor ergonomics which may lead to absenteeism, decreased well-being (e.g., job exhaustion), and even withdrawal (see e.g., [5, 24, 27]).

Against this background, the maintenance of good health, functioning, and work ability among employees and the improvement of working conditions are key areas that organisations and employers need to consider [22, 27]. Several measures and strategies can help organisations with supporting and retaining their ageing workers. Among these, age management has a long tradition in the European Union [36]. It covers multiple human resource measures and practices aimed at sustaining ageing employees’ work ability and helping them to work until retirement age or beyond.

Against the background of rising retirement ages, this study looks at organisations in the HCS in three European countries and seeks to find out whether similar challenges are reported and what measures are taken in order to address these challenges. The study is conducted in HCS organisations (i.e., inpatient and outpatient) by interviewing both employees and their representatives and managers. It offers new information on how ageing employees are supported in the HCS by using different age management practices. Our study addresses several aspects that have not been covered by previous studies. First, this includes the focus on the HCS in three countries. In addition, we conducted interviews with employees as well as various types of managers, which offers a balanced perspective on older employees and age management strategies. Lastly we collected our data at a time when the HCS is under increasing pressure and needs to react accordingly.

### Age management practices supporting work ability and working careers of ageing employees

Age management is based on the idea that organisations should recognise the changes that happen in individuals’ resources (i.e., work ability) in their life course and support them in different age and career phases in order to reach organisational goals. Age management has addressed this by combining the work ability of personnel with strategical human relation management for reaching organisational goals. Thus, age management means, according to Ilmarinen [23], ‘managing the work ability and organisation of work from the viewpoint of the life course and resources of people whether the changes are caused by the ageing process or by other age-related factors’. It considers age-related factors in daily leadership and management and enhances individual resources, and adjusts the work tasks to accommodate and utilise individuals’ abilities.

**Fig. 1:**
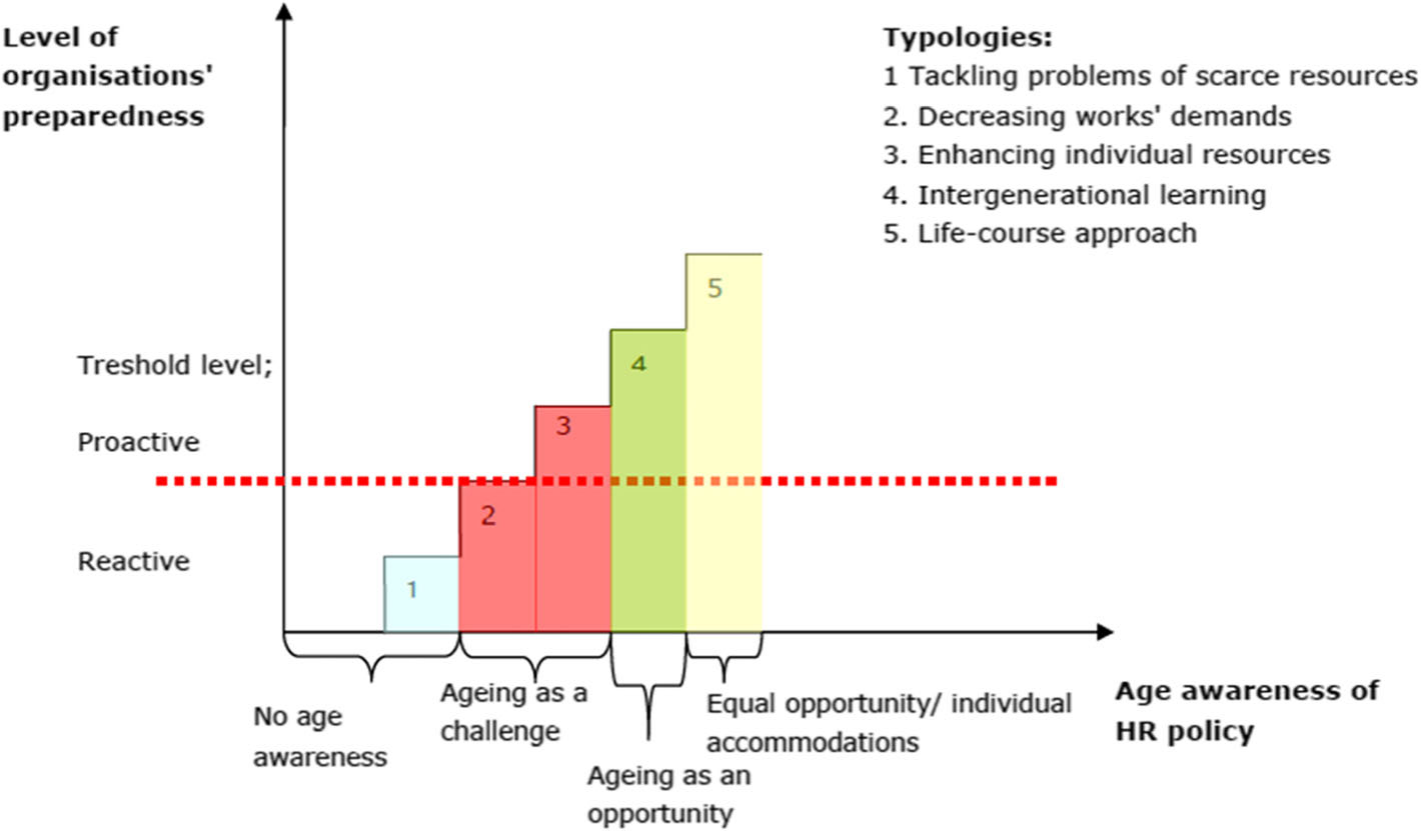
The typology of age management practices ([38], p. 21). [no color in print needed]

Wallin and Hussi [38] have developed a practical assessment tool of age management practices in organisations relevant to the current study. The tool has been created on the basis of the European database of best practices in age management covering 114 cases across the EU member states (see [10] EuroFound), which was itself an expansion of Walker and Taylor’s [37] original work on age management. Wallin and Hussi found that age management practices implemented in organisations differed in two dimensions: 1) age awareness of human relation (HR) policy and 2) level of organisations’ preparedness in age management measures. The first dimension varies from no age awareness, to seeing ageing as an opportunity, and finally to providing equal opportunities, whereas the second dimension varies from reactive to proactive measures. By applying these two dimensions, five typologies of age management practices were identified (see Fig. 1). The authors add that: “It has to be noted that the hierarchical structures do not indicate order of superiority, but rather the temporal maturity.” ([38]: 21).

The first typology called tackling problems of scarce resources is characterised by no prior awareness of age and employee structure and resolving imminent challenges, such as lack of skilled employees. Organisations that have this approach typically respond to labour shortages by recruiting their former retired or unemployed employees and modifying the work requirements with narrow methods to correspond with the work ability of the older employees. However, long-term planning is missing and the organisations are forced to develop novel solutions to acute problems. The organisations that belong to the second typology, decreasing work demands, have better awareness of their age structure. However, they see ageing as a challenge and are especially concerned with the risks related to older employees (e.g., increased labour costs related to sickness absences and disability pensions, a potential threat for productivity). They aim to address these concerns by decreasing job demands. This is done for example by work time/shift arrangements. These measures are mainly reactive and thus implemented after the work ability of employees is already weakened. Moreover, the typology does not take into account the positive resources of older employees as is done in the third typology of enhancing individual resources. This typology resembles the second one in an assumption that older employees are more vulnerable than younger ones, and that ageing brings along challenges and problems. However, this typology aims also to enhance employees’ resources and at the same time modify tasks according to the ageing employees’ work ability, and not just decrease work demands. The perspective is thus more proactive than reactive. The fourth typology labelled intergenerational learning sees ageing as an opportunity and uses proactive age management measures. Age management is integrated into organisations’ practices and personnel policies and they communicate positive age attitudes in all their measures. The organisations are also aware of demographic changes and appreciate the positive aspects of older workers including experience, tacit knowledge and skilful performance. The last typology of life-course approach emphasizes equal opportunities for everyone instead of age-specific measures. Thus, the HR measures are available for all employees regardless of their age and the organisations support work ability of the whole personnel. The HR practices are proactive and the organisations try to avoid and prevent problems in advance; employees are encouraged to do the same (i.e., take care of their personal resources such as competence).

To our knowledge, only few studies have analysed age management approaches within the HCS: Fuertes et al. (2013) carried out case studies on age management in small and medium sized enterprises in different sectors, including the HCS. They found evidence of some stereotypes, e.g. that ageing workers were viewed as not suitable for the work due to its physical demands. Moreover, the authors found that most of the training was mandatory, and there was a view that the ageing workers are the less keen to engage with it. In a European project, Baldauf and Lindley (2013) collected 15 studies in social care from Germany, Denmark, France, Italy, the Netherlands, and Poland (see also [15]). In terms of hiring and recruitment, they found that age management either involved non-discriminatory hiring or in some cases involved hiring ageing workers. Age un-specific health promotion was also common, e.g. with respect to moving and handling equipment. Moreover, they found that flexibility was common, allowing workers to move to part-time work, or otherwise find individual solutions with managers.

Besides age management practices, there are several studies presenting and categorizing different HR practices for ageing workers. Kooij et al. [28] have summarised the previous HR literature into four bundles of HR practices for aging employees based on the lifespan theory of selection, optimisation and compensation [2]. The four clusters of practices are: development (e.g., career planning, mentoring), maintenance (e.g., performance pay, health check-ups), utilization (e.g., lateral job movement), and accommodation (e.g., early retirement, job crafting). Taneva, Arnold, and Nicolson [33] conducted a qualitative analysis of the experiences of ageing workers in two countries and two sectors, with the HCS being one of these. They found that older workers see their career more in terms of development than decline and identified nine different types of organisational support that were perceived as most beneficial. These nine types of support covering different measures of age management were work (1) meaningfulness, (2) social cohesion, (3) knowledge transfer, (4) feedback, (5) recognition, respect and voice, (6) compensation and benefits, (7) work-life balance, (8) job control, and (9) learning and development. More specifically, learning and development for example, refers to having equal access to learning and development opportunities, which could cover measures such as training and qualification, regardless of the age of the employee. Work life balance includes measures such as reduced hours or part-time work patterns. Moreover, the authors developed a multi-level model of older workers’ conceptualisations of age differences at work, which covers four main themes (older vs younger workers, age related challenges, challenges in late career, and work approach).

Comparing these studies and previous age management literature, it becomes clear that there are different approaches in conceptualising age management strategies and age-related HR practices. As we mainly take an organisational age management perspective with a direct focus on ageing employees and measures supporting work ability, we use the typology by Wallin and Hussi, which also acknowledges challenges that organisations are facing. Nevertheless, we take an inductive approach rather than pose any specific hypotheses regarding the measures applied in our case organisations.

## Methods

### Design

With the aim of seeing whether similar challenges in the HCS are reported across the three sample countries and to explore how age management practices are applied by organisations in this sector, we followed a multiple case study design ([39] Yin, 2019), with organisations being the unit of analysis. The cases were sampled purposefully across all three countries (Germany, Finland, and the UK). Those countries were chosen for multiple reasons: Firstly, they face similar challenges with respect to HCS (see introduction). Secondly, they have a similar economic background and a tradition of age management and hence comparable experiences. Thirdly, in all countries there are discussions on increasing the retirement age ([19] Hess, 2016 [26];).

### Case selection and data gathering

The organisations were recruited using established contacts from previous projects; participants were recruited following a nonprobability sampling strategy through announcements within their organisations. The only inclusion criterion for employees was being involved in care work, which includes professional carers but also care assistants. All interviews were carried out between 2017 and 2018; all of them face-to-face on the premises of the participants’ organisations. The researcher in each country approached all organisations, conducted the interviews and analysed the data. There were no restrictions with respect to the size of the organisation or ownership. An information leaflet about the purpose and structure of the interview was given to potential participants and informed consent was gained. All interviews were audio recorded and transcribed verbatim; anonymity and voluntariness were ensured to participants and organisations. The interviews were conducted in the national language (German, Finnish, and English) and all quotes provided in the results were translated if necessary.

We carried out semi-structured interviews on-site with employees as well as representatives of management (Additional files 1 and 2). The employees interviewed were care workers/nurses (certified as well as nursing assistants) with different tasks. All of them were or had been involved in care work. A topic guide was developed in three languages (English, Finnish, and German). The guide was based on the literature (see section ‘background’) and covered several themes: organisational background and structure; current challenges in the HCS from an organisational perspective, such as potential consequences of prolonging working lives; implemented age management measures including their aims and impact.

### Data analysis

Our main interest was on the perceptions and experiences of individuals. The data were analysed using qualitative content analysis [25, 30]. Prior to coding, the material was anonymised. Any information that could lead to identification of the respondent was removed, including names and locations. The process was organised according to the following steps. At first there was a familiarisation with the data, including reading of each manuscript multiple times. Afterwards, categories were generated following a deductive-inductive approach. This means that we generated categories based on previous research. In addition, we developed categories based on the interviews. Therefore, two researches, who also conducted the interviews, independently worked through approximately 25% of the data and discussed the results. The next step was the first coding phase of the data; the researchers who developed the categories went through approximately 50% of the material. If necessary, the coding scheme was refined; disagreements were solved through discussion. Using the final codebook, the interviews were analysed manually or with software support (MAXQDA12). The analysis of the gathered data was undertaken by the researcher who conducted the interviews. Our aim was not to quantify themes and results but to explore perceptions within the sector ([9] Denscombe, 2007).

## Results

In total, we conducted eleven case studies, six in Germany, two in the UK, and three in Finland, comprising 54 interviews. About half of them were private and half-public or non-profit. The organisations were female-dominated, with only ten male interviewees. Employees’ mean age was approximately 47 years, ranging from 24 to 63 years. The background characteristics of the study participants showed that the participants representing employees as well as the managers had very diverse backgrounds. The participants’ length of employment with their current employer was on average 12.4 years. Table 2 presents the background characteristics of the case organisations. The number of interviews varied between case organisations and on average interviews lasted for 35 min.

**Table 2:** Overview of organisations

Case	Number of interviews	Number of employees	Ownership	Organisation type	Workforce characteristics
FIN1	3	150–200	Private	Occupational health	Female (90%), part time (25%), permanent contract (98%), 48 years mean age
FIN2	3	350	Public	Hospital	Female (95%), part time (11%), permanent contract (77%), 45 years mean age
FIN3	3	2.000	Municipal	Municipal health care organisation	Female (91%), part time (13%), permanent contract (75%), 44 years mean age
UK1	8	400	Non-profit organisation	Social housing and care	Predominantly female
UK2	8	460	Non-profit organisation	Inpatient care facility	Female (75%), part time (75%), 43 years mean age
GER1	7	250	Private	Inpatient care facility	Predominantly female
GER2	5	900	Municipal	Inpatient care facility	Female (90%), older than 50 (25%)
GER3	8	200	Private	Hospital	Predominantly female
GER4	4	1.600	University hospital	Hospital	Female (majority), 43 years mean age
GER5	2	54	Private	Outpatient care	nn
GER6	3	40	Private	Inpatient care facility	Predominantly female

We first describe the perceived challenges in each organisation. Then, we focus on the organisational measures applied. They will be analysed drawing on the typology by Wallin and Hussi [38]. Table 3 shows the main challenges that were reported in each organisation and the age management measures which have been implemented.

**Table 3:** Typology of age management practices

Case	Challenge						Age management measures				
	Lack of skilled personnel	Difficulty to recruit	Ageing workforce	Short term substitutes	Staff turnover	Demanding working conditions	tackling problems of scarce resources	decreasing work demands	enhancing individual resources	intergenerational learning	life-course approach
FIN1		X	X			X	X	X	X	X	
FIN2			X	X		X	X	X	X		
FIN3				X		X	X	X	X		
UK1	X	X			X	**X**	X	X	X		X
UK2	X	X				X	X	X			
GER1	X				X	X	X				
GER2					X	X	X	X	X	X	X
GER3	X					X	X				
GER4	X					X	X	X			
GER5	X	X				X	X	X			
GER6	X	X				X	X	X			

### Current challenges within the HCS

The case studies indicated several common challenges within the HCS but also differences between the countries. The most dominant challenge highlighted in Germany was the shortage of skilled care workers despite many people being encouraged to start working in the HCS given vacancy rates:

Today you have a lot of people who are sent by the employment office and there are always one or two areas where the employment office says, “We always need people there”. And among other things, it is care for the elderly. (GER, female, 23, employee).

The participants reported that an issue directly related to the challenge of finding skilled personnel is the comparably high turnover. According to the interviewees, the lack of skilled personnel, and the high fluctuation, mainly results from demanding working conditions (e.g., high work load and time pressure) in both physical and mental terms. The demanding conditions also affect whether employees in the HCS think they are able to work until the official retirement age, or even beyond:

At some point you are either completely burned out physically or psychologically. For example, I love this job, I feel predestined for this job, but until 67 I think there, until then my batteries are completely empty. (GER, female, 44, employee).

Moreover, it was mentioned that there are currently not enough substitutes available for short-term absences. According to one interviewee, many people who want to start or who just started working in the HCS drop out.

So a lot of young people introduce themselves. They volunteer and afterwards they do not come again. Or there are those who enjoy it in the beginning and then they see how hard this profession is in itself and then they go into permanent illness, that is [..] you still have them on the duty roster, because there is no other way [..] yes then the problem is that the coverage, the personnel coverage is not given and the rest of the staff have to take a bath in that sense. That is what annoys me so much. (GER, female, 33, employee).

Demanding working conditions were also mentioned in the UK and Finland. These demanding conditions are exacerbated by several trends, including a growing tendency to reduce institutional care in hospitals and nursery homes and increase home help services. At the same time, residents are on average older than in the past and in need of more assistance. Accordingly, employees working in patients’ homes confront more challenges related to work ergonomics and the availability of collegial and managerial support. One of the managers explained:

You have to have certain criteria to now meet that criteria to have full-time, 24-h care. And that criteria is – for want of a better … is the dependency levels have changed dramatically. So we’re looking after people who are no longer mobile, we’re looking after a lot more people have got diagnoses of dementia, Alzheimer’s and things. So the job is physically becoming more demanding and very more mentally demanding as well, you know … very, very mentally exhausting. (UK, female, 45, manager).

Another aspect mentioned in the interviews was the negative image and low acknowledgement of care work and the comparably low payment (specifically Germany and the UK) and the fact that care work is not valued:

And let’s face it, it’s not the best pay in the world. You don’t do care for the money. You don’t. You’ve got a vocation, I always think, because it’s not the best paid job because again, the red tape and the funding and the things like that, you know. We try to … [organisation] is very good in trying to ensure that we recognise staff and we pay above the minimum wage, and try and pay as much as we possibly can, but there’s only so much you can do with a pot of money. (UK, female, 45, employee).

I simply noticed that the appreciation is a very big issue. Not only here in this institution but also in other institutions that I have got to know up to now. (GER, female, 33, employee).

One interviewee also mentioned the payment gap between fully trained and untrained personnel as one reasons for employees leaving care organisations:

I think the younger generation … I have to say if younger people come into it, people are always going to move on for career. NHS pays far better wages than [non-for-profit] care providers. For support workers, you know, for their unqualified support workers, three, four, five pound an hour, more sometimes, you know, in some of the NHS things, so yeah, people are going to move on for that, and then again, people will move on, they want career changes and things like that, you know. (Female, 45, manager, UK).

These factors were mainly mentioned in interviews carried out in the UK and Germany. In Finland, the interviewees were concerned about the lack of skilled employees regarding a specific area of specialization (e.g., occupational health care). However, challenges in recruiting personnel were expected to increase in the near future due to forthcoming ‘retirement boom’. The Finnish informants emphasised that their organisations should be better prepared for this. On the other hand, they highlighted that health care organisations recruited their former employees once they had reached their retirement age in order to balance both temporary and permanent labour shortages.

### Organisational strategies in dealing with current challenges in the HCS

Taking a closer look at the age management measures that were implemented, we identified a broad variety of practices across the cases. The results showed that measures supporting the work ability of (ageing) workers encompassed several dimensions such as recruitment, training, career development, flexible working practices, health promotion, redeployment, employment exit, or comprehensive approaches.

The results showed that many organisational measures aimed at decreasing job demands and were thus accommodative. The most widely used measures related to work time arrangements. Reduced working hours and work shift arrangements were commonly used in all countries, and job alternation leave were additionally used in the Finnish and German organisations. These measures were seen to enhance employees’ recovery if the work itself or the life situation of the employee were demanding.

The UK case studies highlighted the fact that night shifts are necessary in the sector as “care is a 24 hour business”. One employee had an opinion that ageing workers sometime prefer shift work because nights are less busy with residents being asleep:

There’s quite a few older people and I think when it gets … I mean obviously it’s a stressful job, physically and mentally … I think staff tend to swap shifts and start going to nights. I’m not saying it’s not as stressful it’s not as busy, you know what I mean? Obviously you’re not dealing with families and management and stuff” (Female, 61, employee).

The Finnish informants perceived that sometimes the employees do not utilise these arrangements, although the arrangements would benefit their work ability. One reason for this was described to be the negative effects of the arrangements on employees’ current income or future pension. In some Finnish cases, the employees were even allowed to plan their work shifts independently by following the jointly agreed principles. In this way, the employees obtained more control over their working hours. In the UK, one respondent reported the need to be strategical in asking for flexible working, making sure it fit in with the company, stating “you have to be quite firm or they mess you around” (Female, 60, employee, UK). From an inequalities perspective, this suggests flexible working arrangements should be democratised to remove the need for strategical requests, which some employees are likely to be more adept at that others.

The Finnish informants described their organisations’ career promotion and training opportunities as equally open for everyone, which was also mentioned in interviews in Germany. However, ageing employees were sometimes perceived to be less interested in promotions and prefer their current positions. Despite an equal amount of money allocated for training employees of different ages, the training was argued to be used more actively by the younger than older employees. In the UK there was also a sense that most training was in response to statutory requirements rather than offering genuine opportunities for learning:

You know, I would say the training, as time’s gone on, has been less person centred. It’s been … it’s cost … You need first aid, you need health and safety, you need lone working risks. You know, you need your basic ones. So, when you’re saying, oh I fancy learning about so and so, I could apply that to this job, that’s not been as good the last few years. (UK, female, 60, employee).

In the UK, training in the form of eLearning was common. Workers were generally negative about this, preferring face-to-face contact instead. This was echoed by the views of management, and, in Germany also by colleagues, who reported ageing employees are not used to using digital technology and hence have problems with changes, in particular if ICT is involved.

I sometimes think that it shouldn’t be underestimated that change for older people is quite…can be quite traumatic for them and we live in a very fast paced changing environment and I know it’s an old saying, but it’s difficult to train an old dog new tricks, but it really is difficult to, because, you know, we’re changing all the time, it’s very fast paced change, we change the processes we use, we change the technology we use on different things, we use different IT systems, we’re looking at a different one again. (UK, male, 58, manager).

With respect to measures related to knowledge transfer, the case organisations did not report hiring new employees before experienced ones retire. In addition, mentoring was not used consistently. However, a common approach was to rehire workers who had retired or offering them to work on a limited basis once they were about to retire. There were mixed views expressed by the employees. While some felt that this was a good opportunity, others could not imagine working longer then they have to. Moreover, some interviewees felt that they need to work longer in order to avoid pension cuts. Some organisations recruited their former employees with part-time contracts once they had reached their retirement age. In all cases there were no particular strategies to recruit younger employees. In fact, in many cases age was not seen as an important recruiting criterion. In Finland, the case organisations emphasized the importance of former employees in balancing both temporary and permanent labour shortages:

We have our own substitute unit called […] and we encourage our retired employees with good physical condition to make a contract with […] and continue their working career as a substitute (FIN, female, 62, manager).

For health promotion the case organisations offered healthcare services and check-ups for each employee and enhanced physically active lifestyles by offering subsidised exercise activities. In the UK this was extensive in one organisation, but not mentioned in another. In the former, it included discounts on gym membership, links with a private healthcare provider, rebates on dentist or optician fees, massages, counselling/therapy, and in terms of the work environment supplying different chairs if necessary. Moreover, the Finnish organisations took steps to intervene in employees’ sickness absences early on. Accordingly, supervisors were obliged to have a discussion with the employee and occupational health care services when the sickness absences of an employee exceeded a certain amount (e.g., 30 days).

Although some measures were proactive, most of the measures were used reactively after employees’ work ability had already weakened. For example, the measures related to job modification (i.e., modification of tasks, work time arrangements) have been used reactively in many cases. Accordingly, in some cases employees’ tasks were changed to be less demanding by replacing some part of clinical work with office work. Redeployment had also helped to bring those employees back to work who had been on long and expensive sickness leaves due to impaired work ability.

In most cases, the implemented measures were not evaluated afterwards. In Finland, the informants from public organisations stated that many measures had negatively affected the cost of early disability pensions. Moreover, they stated that the individually tailored measures had proved to be the most effective.

If a supervisor notices that an aging employee would benefit for shorter hours or should receive some extra support, for instance…the applied measures should be tailored to the specific needs of this employee… not just offer the same measures for everyone in this age group…. …in this way the results would be better (FIN, female, 49, employee).

Applying the typology by Wallin & Hussi [38], most of the organisations in our sample can be classified as type 1, ‘tackling problems of scarce resources’, and type 2, ‘decreasing work demands’, as most of the organisations did not have age awareness or they saw ageing as a challenge. Moreover, they used more reactive than proactive measures which is typical for organisation of type 2. Table 3 provides an overview of our sample and the different types as well as the challenges that were reported.

Comparing the types of organisational support with the challenges that were described by the interviewees as most pressing, we found that the organisations in our sample focussed mainly on two or three measures, such as shift rotation, or health promotion. However, in most cases these measures did not have any focus on ageing workers. Hence, the majority of organisations in our sample can be characterised as having either no age awareness or low age awareness.

## Discussion

We found variations in age management approaches across both organisations and countries. In Germany, the biggest challenge reported was difficulties in finding new suitably qualified employees. Focusing on ageing workers could be one way in overcoming this shortage: recruiting ageing workers could be a strategy in tackling the lack of skilled workers as they have a high level of experience and are familiar with the sector and its characteristics. Although the interviewees felt that the difficulty in finding new staff will increase in the future, they felt there is a lack of attention to this issue now. One explanation could be that organisations exclusively focus on current problems in hiring new staff or on what they see as more strategically important issues (e.g., health care reform in Finland), which does not allow space for forward planning with respect to the ageing workforce. Furthermore, it is not only difficult to find new personnel, but also to retain existing personnel. As well as recruiting or retaining ageing workers, one strategy might be for companies to look abroad for new personnel – but with limited success according to our interviews. Otherwise, younger workers need to be trained. Again, this bears risks as our interviews indicated that young employees are motivated to withdraw from core care tasks, or find higher paying jobs elsewhere.

The most prominent measures mentioned by the interviewees were reducing hours or part-time work patterns, which have been implemented by nearly all organisations in our sample. The main reason for this is to avoid redundancies and early workforce exit in an aging workforce [8]. However, one could argue that these measures are more or less standard within the sector. The same is true for flexible working schemes, at least if this covers working time. Only very few organisations offered flexible working schemes in terms of task variety. Offering the possibility to vary tasks could be a way to support (ageing) workers as this provides the chance to recover. While nearly all organisations studied had implemented one or two measures, most related to health promotion and flexible working practices. Strategies targeting multiple dimensions were missing and did not cover the work environment.

Given the shortage of skilled personnel, employers are under pressure and need to implement HR-measures in order to keep their staff and attract new ones. The manager of one organisation in our sample, which can be classified as the type five, ‘life-course’ approach according to the typology by Wallin and Hussi, specifically sees the focus on all employees, regardless of their age, as a strategic advantage. It helps not only in recruiting new employees, but also in attracting personnel from competitors. On the other hand, employees have more power as they will easily find a new job. Consequently, they can apply pressure to their employers and negotiate better working conditions. We found that this seems rarely to be the case, however. More typical were individual strategies like applying for administration jobs such as shift management.

Extending working lives bears several risks in the HCS. Due to its demanding working conditions and the low payment, the preferred retirement age is lower compared to other sectors (Mäcken et al., 2018), which could result in potential inequalities as not all will benefit equally from implemented measures. In many cases almost all measures were targeted at the whole workforce, i.e., not only those of a certain age or those with declined work ability. Nevertheless, the informants argued that some employees were perhaps more privileged to use the measures. For example, employees with a better economic situation and a higher qualification level have better chances to reduce their working hours. In addition, it was mentioned that sometimes the operation of the work unit/department did not allow applying all the measures that would have benefitted the employees the most.

In order to respond to these challenges, a life course approach to age management offers multiple advantages. As life course management emphasises career phases and life situations of individual employees more than their age in supporting their work ability, it might also diminish the negative stereotypes related to ageing employees [35].

Besides organisational strategies, we also found individual ways in dealing with the challenges named. On the individual level, a common strategy in reacting to the demanding working conditions was found in further training, qualification, and professionalisation to withdraw from ‘core’ care work to administrative tasks such as shift management. This strategy was also followed by some of the younger employees in our sample that have been working in the HCS only for a couple of years. However, nearly all interviewees were aware that not everyone will be able to work in one of these jobs as they are limited. Another strategy reported by one of the interviewees was to reduce working time and to work for an external company in the HCS on a limited financial basis. The main motivation was to be more flexible and to have more leisure time with only minor financial drawbacks.

Interestingly, we did not find any evidence on assistive technology. While there are several debates on this topic (cf. [31]), this was not mentioned by the interviewees. An explanation could be that such technologies have not yet scaled up and are not used in the sector on a widespread basis.

### Strengths and limitations

Our study has several strengths, but also limitations. With regard to strengths, we compare a large dataset of eleven organisations within the HCS in three countries with 54 individual interviews in total. Compared to similar studies [15] we included both managers’ and employees’ views, and integrate organisational and individual perspectives. On the other hand, we did not conduct the same number of case studies and/or interviews in each country making it more difficult to compare the results. In addition, a comparison of the organisations in a more detailed way would be helpful. In order to do so, the sample should contain a sufficient number of organisations of different type (e.g. type of care institution, size, etc.). Nonetheless, we argue that the organisations were sufficiently similar – all involving health and social care in some capacity – for common issues to emerge. Future studies should take this into account and see if there are specific differences, e.g. by comparing outpatient care organisations.

### Recommendations

Organisations/ companies in the HCS should consider investing in age management. Not only because it makes them more attractive to potential employees but also to keep their staff – in particular, in times when it is difficult to find skilled employees. In this regard, age-management should be understood as life-course management and not focus on certain age groups. However, addressing the needs of ageing employees could also help to deal with the current lack of skilled employees. A recent study in Finland [34] showed that ageing employees are not necessarily aware of different retirement alternatives and economic consequences of them to their future pensions, a finding that our research underlines. Thus, more information and guidance should be offered regarding different options so that more employees are aware of the possibility of a part-time pension for example, though this possibility is country dependent.

## Conclusions

Health and social care work is physically and mentally demanding. Work time arrangements and job modification are needed to help ageing employees to continue working until retirement age. In addition, many of the measures benefit both the employee and the employer/organisation. Employees would have improved occupational well-being and more meaningful careers, whereas employers would have more committed employees with better work ability, productivity and who are more motivated to have a longer career. Age management as a concept offers promising possibilities to increase the work ability of (ageing) workers but only few organisations seem to see ageing as an opportunity; they either have low age awareness or see ageing of their staff as a burden. Against the background of a lack of skilled care workers within the HCS, we found that care organisations focus on younger workers and there is also an increasing trend to hire personnel from abroad. Explicitly focusing on ageing workers could be another approach in dealing with the current lack of personnel.

## Supplementary information

**Additional file 1.** Interview guide management.

**Additional file 2.** Interview guide employees.

## Data Availability

The datasets used for the current study are available from the corresponding author on reasonable request.

## Abbreviations

EU: European Union
HCS: Health and Social Care Sector
HR: human relations
ICT: Information and Communication Technologies
NHS: National Health Service
SME: small and medium sized enterprises
UK: United Kingdom
