# Supplementary material for: Challenges and practices in promoting (ageing) employees working career in the health care sector – case studies from Germany, Finland and the UK
Source: BMC Health Serv Res. 2019 Nov 29;19:918. doi: 10.1186/s12913-019-4655-3 (PMC6884779; doi:10.1186/s12913-019-4655-3)
Supplement: Supplementary file 1 — Additional file 1. Interview guide management. [file 12913_2019_4655_MOESM1_ESM.docx]

**Additional File 1. Interview guide management**

**Interview guideline**

**Background**

- Background of the organisation *(if available)*
  - Location
  - Size (exact number)
  - Legal form
  - Ownership (private, non-profit, municipality)
  - Founded in…
  - Subsidiaries
  - Turnover/sales
  - Workforce structure
    - Age composition/age structure
    - Percentage of female/male workers
    - Percentage of part-time employees
- Background of the interviewee
  - Educational and professional background
  - Experience *(in years)*
  - Role within the organisation
  - Gender
  - Age *(in years)*
  - Years working for the organisation

**Context**

- What are the major strategic issues or challenges facing your organisation at the moment?
- What do you think are the main human resource challenges facing your organisation and why? *(prompt: difficulties recruiting, retaining key staff, particular skillsets, ageing of employees)*
- Can you give me one or two examples of how your organisation is addressing these challenges?
- Could you describe how employees‘ views are taken into account and how the employees are empowered in your organization? *(prompt: Trades Union, staff association, etc.)*
- Nationally the workforce is getting older, with fewer young people entering the workforce, and more in their 50s. How important is the ageing workforce for your organisation? *(prompt: how far is your workforce ageing? Do you have numbers?)*
- Do you think that encouraging older employees to stay in work longer could address the main HR challenges which you face?
  - If yes, please describe in how far and why.
- Do you think that your sector will be affected differently compared to other sectors?
  - If yes, please describe in how far and why.

**Age-management measures – development**

- Have you/your organization implemented any age-management measures/policies/practices?
  - If so, what were the reasons to implement age-management measures? *(prompt: organizational changes, external influences like labour market situation)*
  - If no, what were the reasons not to implement these?
- Could you give me a description of the measure(s) you have implemented? *(prompt: target group [age structure, gender, profession], aims/objectives,* *when these were introduced)*
- What was/were the main theme(s) of these measures? *(prompt: recruitment, training, lifelong learning and knowledge transfer, career development, flexible working practices, health promotion, workplace design)*
- Who was involved in planning/implementing those measures? *(prompt: management, HR, all employees, trade unions, external advisers)*
- Who were the key actors?
- What facilitators/barriers did you/your organisation experience when implementing those practices?
  - *If applicable:* How were the measures modified?
- Have you/your organisation previously tried to implement any measures that were not continued? *(prompt: target group [age structure, gender], aims/objectives, time)*
  - - If yes, what kind of measures?
    - If yes, what were the barriers/the reasons they failed or were not continued?
- Could you describe how employees‘ views are taken into account and how the employees are empowered in your organization considering age-management? *(prompt: a recognised Trades Union, staff association, etc.)*

**Impact and outcomes of age-management measures**

- Did the measure meet their aims and objectives?
  - - If not, what were the reasons?
- Were the effects evaluated?
  - - If yes, how?
    - If not, why not?
- Which occupational fields would gain the most from introducing/ maintaining age management practices?
- Which types of costs did you face when introducing age management practices? *(prompt: Costs of implementation, material costs (equipment costs), training costs, costs of „extra” workforce to adjust for flexibility (e.g. retired employees), organizational costs, estimation in EUR/GBP)*
- What were the effects of the measures? *(prompt: reduced absenteeism, medical savings, reduced employee turnover (hiring costs, knowledge loss), increased employee motivation and satisfaction, sustained focus on “customers” (higher productivity), higher customer satisfaction, more “customers”, better corporate image)*
  - *If applicable:* What are/were the long-term effects?
  - Did/do the benefits outweigh the costs?
    - Why/in how far?

**Closing**
