# Supplementary material for: Challenges and practices in promoting (ageing) employees working career in the health care sector – case studies from Germany, Finland and the UK
Source: BMC Health Serv Res. 2019 Nov 29;19:918. doi: 10.1186/s12913-019-4655-3 (PMC6884779; doi:10.1186/s12913-019-4655-3)
Supplement: Supplementary file 2 — Additional file 2. Interview guide employees. [file 12913_2019_4655_MOESM2_ESM.docx]

- **Additional File 2. Interview guide employees**

**Background**

- Background of the interviewee
  - Educational and professional background
  - Experience *(in years)*
  - Role within the organisation
  - Gender
  - Age *(in years)*
  - Years working for the organisation
  - Could you describe your current job situation? *(prompt: working part time, in shifts, etc.)*

**Context**

- Nationally the workforce is getting older, with fewer young people entering the workforce, and more in their 50s. In how far, do you think, does this affect the organization you are working in? *(prompt: how far is your workforce ageing? Do you have numbers?)*
  - Do you think that managers in your sector generally have strong views about the age range appropriate for particular jobs?
- Do you think that your sector will be affected differently compared to other sectors?
- Does the organisation you are working in have any process for formal consultation with employees? *(prompt: a recognised Trades Union, staff association etc.)*

**Age-management measures**

- Have any age-management measures/policies/practices been implemented in the organization you are working in yet?
- If so, could you give me a description of these measure(s)? *(prompt: target group [age structure, gender, profession], aims/objectives,* *when these were introduced)*
- What was/were the main theme(s) of these measures? *(prompt: recruitment, training, LLL and knowledge transfer, career development, flexible working practices, health promotion, workplace design)*
- Who was involved? *(prompt: management, HR, all employees, trade unions, external advisers)*
  - Who were the key actors?
  - How were the employees involved?
- How do your rate the measures from your personal point?
- Did the measures meet their aims and objectives?
  - If No, what were the reasons?
- What do your colleagues think about these measures?
